# Focused ultrasound enhances targeted curcumin delivery and alleviates behavioral and neuropathological deficits in a Parkinson’s disease mouse model

**DOI:** 10.3389/fnagi.2026.1740256

**Published:** 2026-02-09

**Authors:** Shu-Mei Yang, Sen-Hi Yeoh, Meng-Ting Lin, Ming-Yen Hsiao, Chueh-Hung Wu, Sung-Jan Lin, Wen-Shiang Chen

**Affiliations:** 1 Department of Biomedical Engineering, College of Medicine and College of Engineering, National Taiwan University, Taipei, Taiwan; 2 Department of Physical Medicine and Rehabilitation, National Taiwan University Hospital, College of Medicine, National Taiwan University, Taipei, Taiwan; 3 Department of Physical Medicine and Rehabilitation, National Taiwan University Hospital Hsin-Chu Branch, Hsinchu, Taiwan; 4 Department of Dermatology, National Taiwan University Hospital, Taipei, Taiwan

**Keywords:** anti-inflammatory, blood–brain barrier, curcumin, drug delivery, focused ultrasound, neuroprotection, Parkinson’s disease

## Abstract

**Introduction:**

Curcumin exhibits potent neuroprotective properties but is limited by poor blood-brain barrier (BBB) permeability. This study aimed to evaluate whether focused ultrasound (FUS)-mediated BBB opening enhances curcumin delivery and therapeutic efficacy in a 6-hydroxydopamine (6-OHDA) mouse model of Parkinson’s disease (PD).

**Methods:**

Male C57BL/6 mice with unilateral 6-OHDA lesions received intravenous curcumin with or without FUS targeted to the lesioned striatum for 4 weeks. Behavioral performance was assessed using rotarod and open-field tests. Postmortem analyses included tyrosine hydroxylase (TH) immunostaining in the striatum and substantia nigra pars compacta (SNpc), glial fibrillary acidic protein expression, and fluorescence quantification of curcumin accumulation. Microglial activation and pro-inflammatory cytokine expression were evaluated using ionized calcium-binding adaptor molecule 1 (Iba1) and interleukin-6 (IL-6) staining. Safety was evaluated by histological examination for hemorrhage or necrosis.

**Results:**

Significant improvements in motor coordination and exploratory activity were observed in the FUS + curcumin group, particularly during early stages of degeneration. Histologically, combined treatment was associated with greater preservation of TH-positive dopaminergic neurons in the SNpc and reduced astrocytic activation in the striatum compared with curcumin alone, whereas striatal TH fiber density did not differ between curcumin-treated groups. No significant differences were observed in striatal Iba1 or IL-6 expression across groups at the 4-week time point. Enhanced curcumin accumulation in the brain was observed following FUS, as demonstrated by fluorescence quantification. No tissue damage or adverse effects were observed after repeated sonications.

**Discussion:**

This is the first study to demonstrate the efficacy of unmodified curcumin combined with FUS in a 6-OHDA PD model. The findings support FUS as a safe and effective strategy to transiently disrupt the BBB and enhance the central nervous system delivery of small-molecule therapeutic agents, offering translational potential for regionally targeted, non-invasive treatment of PD.

## 1 Introduction

Parkinson’s disease (PD) is a progressive neurodegenerative disorder that primarily impairs motor function (Sveinbjornsdottir, 2016). PD is characterized by early and prominent degeneration of dopaminergic neurons in the substantia nigra pars compacta (SNpc), which reduces dopamine levels in the striatum through disruption of the nigrostriatal pathway. This pathway is a key component of the basal ganglia circuit that regulates voluntary movement (Watanabe et al., 2024). The resulting dopamine deficiency gives rise to the hallmark motor symptoms of PD, including bradykinesia, resting tremor, rigidity, and postural instability (Chen et al., 2022). Beyond motor impairments, patients with PD often experience non-motor symptoms, including cognitive decline, mood disorders, and autonomic dysfunction, all of which significantly reduce quality of life (Chen et al., 2022). While dopaminergic therapies provide symptomatic relief, no available treatment modifies the underlying neurodegenerative process (Pardo-Moreno et al., 2023), indicating the need for disease-modifying strategies.

Neuroinflammation is increasingly recognized as a central pathophysiological mechanism in PD. Activated astrocytes and microglia drive the chronic release of pro-inflammatory mediators, including tumor necrosis factor-alpha (TNF-α), interleukin-1β (IL-1β), and inducible nitric oxide synthase (iNOS), which in turn intensify oxidative stress, mitochondrial dysfunction, and neuronal apoptosis (Chen et al., 2023; García-Domínguez, 2025). Evidence from both animal models and post-mortem human PD brains shows persistent glial activation, supporting the concept of a sustained inflammatory state that accelerates dopaminergic neuron loss (Chen et al., 2023).

Curcumin, a polyphenolic compound extracted from *Curcuma longa*, demonstrates promising neuroprotective effects in diverse experimental models of neurodegeneration (Genchi et al., 2024). It exerts potent antioxidant, anti-aggregatory, and anti-inflammatory effects, including suppression of nuclear factor-κB (NF-κB) activation, inhibition of pro-inflammatory cytokine release, and promotion of microglial polarization toward an anti-inflammatory phenotype (Liu et al., 2025). Additionally, curcumin attenuates astrocyte activation, thereby limiting glia-mediated neuroinflammation (Liu et al., 2025; Yu et al., 2016). These mechanisms position curcumin as a compelling candidate for modulating PD-associated neuroinflammation and neuronal loss. Despite these promising effects, the clinical translation of curcumin remains restricted by poor water solubility, rapid systemic clearance, and limited permeability across the blood–brain barrier (BBB) (Sohn et al., 2021).

To address these pharmacokinetic barriers, focused ultrasound (FUS) emerges as a non-invasive technique that may transiently and locally open the BBB when applied in combination with circulating microbubbles (Burgess and Hynynen, 2014; Durham et al., 2024). This strategy enables targeted delivery of therapeutic compounds into the brain and has already progressed to early stage clinical trials for neurological diseases (Durham et al., 2024). Beyond enhancing drug penetration, FUS also exerts neuromodulatory and anti-inflammatory effects, which may further strengthen its therapeutic potential (Grewal et al., 2023; Lin et al., 2025; Wu et al., 2024). Recent studies in PD models investigate the use of FUS in combination with nanocarrier-based curcumin formulations to improve delivery across the BBB (Yan et al., 2021; Zhang et al., 2018). These strategies show encouraging improvements in delivery efficiency; however, their dependence on complex carrier systems introduces challenges in formulation development and reduces the feasibility of clinical translation.

Given these considerations, combining FUS with native, unmodified curcumin presents a potentially simpler and more accessible strategy for therapeutic development. Determining whether this strategy may provide meaningful neuroprotective benefits without relying on engineered carriers remains a critical question.

This study addresses this knowledge gap, by employing a 6-hydroxydopamine (6-OHDA) mouse model, which closely mimics the selective nigrostriatal pathology of PD, to evaluate whether FUS-mediated BBB opening enhances the neuroprotective and anti-inflammatory effect of curcumin. This study aims to elucidate the therapeutic potential of FUS-assisted curcumin delivery by evaluating motor performance, dopaminergic neuronal integrity, and glial activation, and to assess its feasibility as a safe and clinically applicable strategy for PD treatment.

## 2 Materials and methods

### 2.1 Animals

All animal procedures were performed following the guidelines for the care and use of laboratory animals established by the Laboratory Animal Center of the National Taiwan University College of Medicine. The study protocol was approved by the Institutional Animal Care and Use Committee (IACUC) of the National Taiwan University College of Medicine (Approval No. 20220496).

Male C57BL/6 mice (body weight, 25–32 g) were obtained from the National Laboratory Animal Center and maintained under standard housing conditions with free access to food and water on a 12-h light/dark cycle. The animals were randomly assigned to groups using a computer-generated sequence.

### 2.2 BBB opening induced by FUS

The FUS sonication system comprised a waveform generator (33500B series, Keysight, California, United States), power amplifier (1040L, Electronics & Innovation, New York, United States), and commercial focused ultrasonic transducer (Model H104, Sonic Concepts, Washington, United States) with an active diameter of 64.0 mm. FUS was employed using this transducer to target the left striatum (0.6 mm anterior and 1.8 mm lateral to the bregma) (Slézia et al., 2023; Wang et al., 2022). The sonication parameters were as follows: a center frequency of 0.5 MHz, pulse repetition frequency (PRF) of 1 Hz, pulse duration of 10 ms (ms), duty cycle of 1%, and total exposure time of 1 min (Liu et al., 2016).

To assess the safety of FUS-induced BBB opening, healthy C57BL/6 mice were randomly assigned to one of three FUS groups, which received input voltages of 20 millivolts peak-to-peak (mVpp, *n* = 3), 30 mVpp (*n* = 3), or 40 mVpp (*n* = 3) on the waveform generator. These inputs yielded corresponding output voltages of 40, 54, and 75 millivolts (mV), producing acoustic pressures of 0.11, 0.15, and 0.28 MPa, respectively. To calibrate the sonication settings, input voltages (mVpp) were generated by the waveform generator and transmitted to the power amplifier, which subsequently produced the output voltages applied to the transducer. The input voltage represented the control signal from the waveform generator, whereas the output voltage corresponded to the amplified driving signal delivered to the transducer. Acoustic pressures were estimated from the output voltages and prior calibration data, and were employed to assess the extent and safety of BBB opening.

BBB opening was assessed through intravenous administration of Evans blue (EB) (Sigma–Aldrich, St. Louis, MO, United States), which extravasates from the vasculature upon BBB disruption. Histological examination with hematoxylin–eosin (H&E) staining was performed to assess potential tissue injury. The optimal FUS parameters were determined by the degree of EB diffusion in conjunction with the absence of histopathological abnormalities.

Each mouse was anesthetized with a mixture of Zoletil 50 (50 mg/kg) and Xylazine (2.3 mg/kg) and positioned prone with the head secured. The scalp fur was shaved, and residual hair was removed with depilatory cream to ensure effective acoustic coupling. The FUS transducer was mounted on a customized stereotaxic adapter for directional sonication and aligned with the target region in the left striatum. Ultrasound coupling gel was applied between the scalp and transducer interface to minimize acoustic impedance and facilitate effective transmission.

Prior to sonication, 10 μL of Sonazoid microbubbles (1.5 × 10^7^/mL; GE Healthcare, Oslo, Norway) were administered via tail vein injection and allowed to circulate for 1 min. Immediately after ultrasound application, 2% EB (6.25 μL/g) was injected intravenously. Sham group mice received microbubble and EB injections but were not exposed to active sonication.

Mice were euthanized 3 h after EB injection by an overdose injection of Zoletil (150 mg/kg) and Xylazine (6.9 mg/kg). Brains were harvested and fixed in 10% formalin overnight. Coronal brain sections encompassing the sonicated region were prepared, and BBB permeability was assessed by visualizing EB extravasation under a fluorescence macroscope (MVX10, Olympus, Tokyo, Japan).

For histological evaluation, tissues were fixed, dehydrated, embedded in paraffin, and coronally sectioned at a thickness of 7μm. Sections encompassing the center of the sonicated region were dewaxed in xylene and rehydrated through a graded ethanol series. H&E staining was performed, and the sections were examined under a light microscope (BX51, Olympus, Tokyo, Japan) to assess histopathological changes associated with BBB disruption, including hemorrhage, edema, and cellular damage.

### 2.3 Evaluation of curcumin brain permeability with and without FUS-mediated BBB opening

To assess curcumin penetration into the brain following FUS-induced BBB disruption, mice were subjected to the sonication protocol described previously. Male C57BL/6 mice underwent FUS targeting the left striatum immediately after intravenous administration of Sonazoid microbubbles. The sonication parameters were as follows: center frequency, 0.5 MHz; PRF, 1 Hz; pulse duration, 10 ms; duty cycle, 1%; total exposure time = 1 min; and peak negative acoustic pressure, 0.15 MPa.

Nine mice were randomly assigned to two groups: the FUS + Curcumin group (*n* = 5), which received curcumin following FUS exposure, and the Curcumin-only group (*n* = 4), which received curcumin without FUS exposure. In both groups, curcumin (Sigma-Aldrich, St. Louis, MO, United States; purity > 99.5%) was dissolved in dimethyl sulfoxide (DMSO), polysorbate 80, and polyethylene glycol 300, then diluted with sterile saline to achieve a final ratio of 1:1:2:6 (DMSO:polysorbate:polyethylene glycol:saline), yielding a concentration suitable for intravenous administration. Mice were injected via the tail vein with curcumin at a dose of 15 mg/kg, administered immediately after FUS in the FUS + Curcumin group or without sonication in the Curcumin-only group.

To assess curcumin accumulation in the brain tissue, mice were euthanized 3 h post-injection. Whole brains were immediately harvested, placed at −20°C, and stored overnight prior to further processing.

For biochemical analysis, frozen brains were homogenized in 300μL of radioimmunoprecipitation assay (RIPA) buffer (Thermo Fisher Scientific, Waltham, MA, United States) using a tissue homogenizer (PowerMasher II, Nippi, Tokyo, Japan). The homogenates were centrifuged at 14,000 rpm for 30 min at 4°C, and the resulting supernatant was collected for curcumin quantification.

The fluorescence intensity of curcumin in brain lysates was measured using a microplate reader (Infinite^®^ M200 PRO, Tecan Group Ltd., Männedorf, Switzerland) with an excitation wavelength of 430nm and an emission wavelength of 509nm, as previously described (Priyadarsini, 2009). Each brain lysate sample was measured in triplicate to ensure accuracy and consistency of the fluorescence readings.

To account for potential differences in tissue yield, fluorescence intensity was normalized to total protein concentration, determined using a protein assay kit (Pierce Bradford Protein Assay Kit, Thermo Fisher Scientific, Waltham, MA, United States). Final curcumin levels were expressed as arbitrary units per milligram of total protein (A.U./mg protein).

### 2.4 6 -OHDA-induced PD mouse model

A unilateral PD mouse model was developed by stereotaxic injection of 6-OHDA (Sigma-Aldrich, St. Louis, MO, United States) into the left striatum. 6-OHDA was dissolved in 0.02% ascorbic acid in sterile saline, and the solution was freshly prepared on ice under light-protected conditions. PD mice received 1μL of 10μg/μL 6-OHDA at a rate of 1μL/min, while those of the control group were administered an equivalent volume of vehicle solution (0.02% ascorbic acid in sterile saline). Injections were performed using a 50μL gastight syringe with a luer tip (1,700 Series, Hamilton Co., Nevada, United States) fitted with a 30G disposable needle and mounted on an automated infusion pump (Pump 11 Elite Series, Harvard Apparatus, Massachusetts, United States) to ensure precision and consistency.

All mice were anesthetized with Zoletil 50 (50 mg/kg) and Xylazine (2.3 mg/kg) before being secured in a stereotaxic frame (Stoelting Quintessential Stereotaxic Injector, Illinois, United States). Furthermore, 30 min prior to injection, each mouse received an intraperitoneal injection of a 10 mL/kg solution containing desipramine (2.5 mg/mL) and pargyline hydrochloride (0.5 mg/mL) to enhance the selectivity and efficacy of 6-OHDA lesions (Cui et al., 2024; Thiele et al., 2012).

Following induction of anesthesia, the scalp was shaved and disinfected with alcohol swabs. A midline incision was made to expose the skull, and the periosteum was carefully removed. The injection site was localized at the following coordinates relative to the bregma: anteroposterior (AP) + 0.6mm, mediolateral (ML) + 1.8mm, and dorsoventral (DV) −2.5mm (Slézia et al., 2023). After injection, the needle was retained in place for 5 min to allow complete diffusion before being slowly retracted from the injection site to prevent reflux of the injectate.

The 6-OHDA lesion model was verified before treatment initiation. Significantly reduced motor performance was observed in 6-OHDA–lesioned mice compared with the sham group 1 week after injection, as assessed by rotarod testing. Dopaminergic terminal loss in the ipsilateral striatum was further confirmed by tyrosine hydroxylase (TH) immunostaining in a subset of mice, demonstrating the reliability and consistency of the model before FUS–mediated intervention. Lesion establishment was verified prior to treatment initiation based on motor performance deficits. Mice were randomized into experimental groups only after confirmation of lesion-induced impairment. To minimize inter-animal variability, striatal TH immunoreactivity was quantified as the percentage of the ipsilateral striatum relative to the contralateral side within the same animal.

### 2.5 Therapeutic evaluation of FUS-enhanced curcumin delivery in a PD mouse model

**FIGURE 1:**
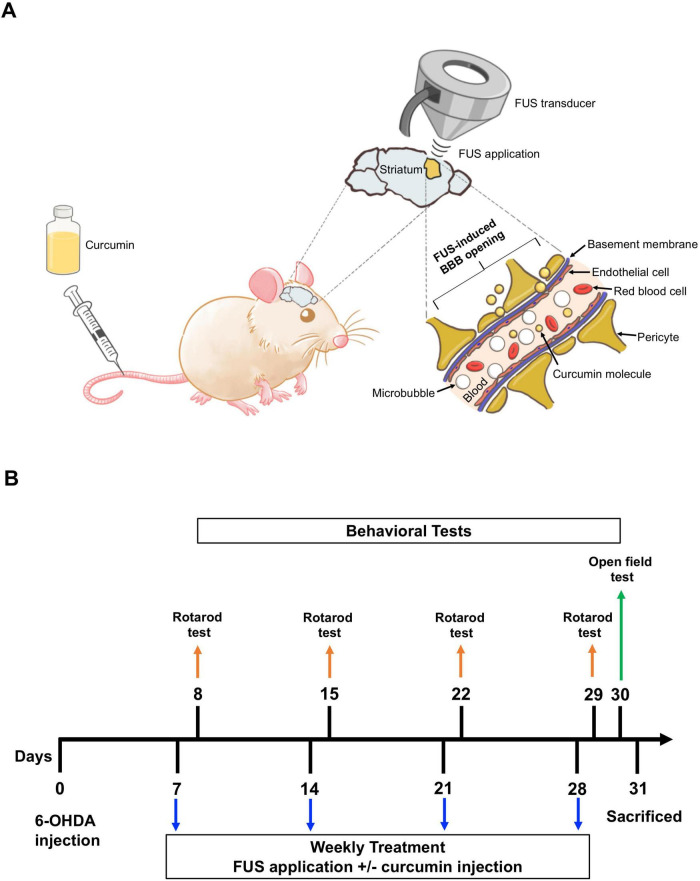
Schematic overview of the experimental design and treatment timeline. **(A)** Schematic representation of the experimental procedure involving focused ultrasound (FUS). Mice received intravenous injection of microbubbles followed by FUS targeting the left striatum to transiently open the blood–brain barrier. Curcumin was administered immediately after each sonication session to enhance brain delivery. **(B)** Timeline of the experimental procedures. The study included four groups: the Sham group, the 6-OHDA group, the 6-OHDA + FUS group, the 6-OHDA + Curcumin group, and the 6-OHDA + FUS + Curcumin group. Treatments began 1 week after lesion induction and were administered once per week for four consecutive weeks. Rotarod testing was conducted weekly to assess motor coordination, and the open field test was performed in the fourth week. Body weight was recorded weekly. Mice were sacrificed 24 h after the final behavioral test for brain tissue collection and subsequent histological and immunofluorescence analyses (blue arrows indicate weekly FUS application and/or curcumin administration; orange arrows indicate weekly rotarod testing; green arrow indicates the open field test).

Figure 1A shows the experimental design. Following a 2-week acclimatization period, 40 male C57BL/6 mice were randomly assigned to four groups: (1) Sham (*n* = 8), (2) 6-OHDA (*n* = 9), (3) 6-OHDA + FUS (*n* = 5), (4) 6-OHDA + Curcumin (*n* = 9), and (5) 6-OHDA + FUS + Curcumin (*n* = 9). Unilateral Parkinsonian lesions were induced by stereotaxic injection of 6-OHDA into the left striatum, as previously described. The Sham group underwent identical surgical procedures but received vehicle injections (0.02% ascorbic acid in sterile saline) instead of 6-OHDA.

Treatment was initiated 1 week after 6-OHDA lesioning. Curcumin (15mg/kg) was administered via tail vein injection once weekly for 4 consecutive weeks. In the 6-OHDA + FUS + Curcumin group, 10 μL of Sonazoid microbubbles (1.5 × 10^7^/mL) were intravenously injected immediately before each sonication session. Following a 1-min circulation period, FUS was applied to the left striatum using the previously described parameters (center frequency, 0.5 MHz; PRF, 1 Hz; pulse duration, 10 ms; duty cycle, 1%; total exposure time, 1 min; and peak negative acoustic pressure, 0.15 MPa). Curcumin was administered immediately following each FUS session to enhance its penetration through the transiently opened BBB. To ensure procedural consistency, mice in the Sham and 6-OHDA groups underwent placebo sonication with the ultrasound probe positioned but without power output, followed by intravenous injection of sterile saline. All groups received four treatment sessions over the 4-week study period.

Behavioral assessments were performed to evaluate therapeutic effects. The rotarod test was conducted once per week for 4 weeks to assess motor coordination and balance, whereas the open field test was performed in the 4th week to evaluate spontaneous locomotor activity and exploratory behavior.

Body weight was monitored weekly to evaluate general health and identify potential treatment-related adverse effects. Furthermore, 24 h after the final behavioral assessment, the mice were euthanized, and their brains were harvested for immunohistochemistry and immunofluorescence analyses to assess dopaminergic neuron survival and neuroinflammatory responses. TH was used as a marker for dopaminergic neurons, while glial fibrillary acidic protein (GFAP) was employed to assess astrocyte activation. Figure 1B shows the schedule of interventions, FUS application, and behavioral testing.

### 2.6 Behavior analysis

#### 2.6.1 Rotarod test

The rotarod test was performed once per week for 4 weeks to assess motor coordination and balance (Rozas et al., 1998). Prior to testing, mice were acclimated to the testing room for at least 30 min. Following habituation, they were trained on the rod at a low rotation speed of 4 rpm for 1 min to familiarize them with the apparatus (Breznik et al., 2024). During testing, the rod rotation speed increased gradually from 4 to 40 rpm over 180 s, and the latency to fall was recorded (Slézia et al., 2023). Each mouse completed three trials per session, and the mean latency to fall across trials was employed for analysis. A minimum inter-trial interval of 15 min was provided to prevent fatigue. This testing design, including brief pre-test familiarization and limited trial numbers, was used to reduce potential learning-related effects across repeated sessions.

#### 2.6.2 Open field test

The open field test was performed in the 4th week to assess spontaneous locomotor activity and exploratory behavior. The testing apparatus consisted of a white polyvinyl chloride box (40 × 40 cm) with a central zone measuring 20 × 20 cm. At the beginning of each trial, mice were placed in the center of the arena, and their movements were recorded for 10 min utilizing the EthoVision XT tracking system (Version 17, Noldus Information Technology, Wageningen, Netherlands). The arena was cleaned with 70% ethanol and dried thoroughly between trials to eliminate residual odors. Parameters analyzed included total distance traveled, reflecting overall locomotor activity; average speed, indicating movement velocity; immobilization time, representing the cumulative duration of immobility; and the number of zone transitions, defined as the frequency of entries from the peripheral zone into the central zone, which is interpreted as an index of exploratory behavior and anxiety-related responses.

### 2.7 Immunohistochemistry and immunofluorescence analyses

#### 2.7.1 TH immunohistochemistry analysis

Mouse brains were harvested the day after completion of the open field test and fixed overnight in 10% formalin. Tissues were subsequently embedded in paraffin and sectioned at a thickness of 7μm.

For TH immunohistochemistry, sections were blocked with 5% bovine serum albumin for 1 h at room temperature, followed by overnight incubation at 4°C with a primary antibody against TH (mouse monoclonal, 1:100 dilution; Genetex, CA, United States). After rinsing with phosphate-buffered saline containing Tween 20 (PBST), the sections were incubated with a secondary antibody (horse anti-mouse IgG; Vector Laboratories, CA, United States) for 3 h at room temperature. Signal amplification was performed using the VECTASTAIN^®^ Elite^®^ ABC Kit (Vector Laboratories, Newark, CA, United States), and immunoreactivity was visualized with 3,3′-diaminobenzidine (DAB) substrate solution. Images were captured using an optical microscope (BX51, Olympus, Tokyo, Japan).

TH immunoreactivity was evaluated separately in the striatum and the SNpc, using region-specific quantification approaches that account for their distinct anatomical and pathological characteristics. For striatal analysis, coronal sections encompassing the central striatum (anteroposterior levels approximately AP 0 to + 0.5 mm relative to bregma) were selected. In the striatum, dopaminergic pathology is primarily characterized by loss of dopaminergic fibers and terminals rather than discrete neuronal somata. Therefore, striatal TH immunoreactivity was quantified as the optical density of TH-positive fibers within anatomically defined regions of interest (ROIs). Optical density values were normalized to the corresponding contralateral, non-lesioned striatum within the same animal and expressed as a percentage. For SNpc analysis, coronal sections encompassing the SNpc (anteroposterior levels approximately AP −2.8 to −3.40 mm from bregma) were analyzed. Given that dopaminergic neurons in the SNpc are organized as discrete cell bodies, TH immunoreactivity in this region was quantified as the number of TH-positive neurons within the defined SNpc ROIs. Cell counts from the lesioned side were normalized to the contralateral side and expressed as percentages.

In both regions, three consecutive coronal sections were analyzed per animal. ROIs were defined manually according to anatomically established boundaries based on a standard stereotaxic mouse brain atlas and were applied consistently across all animals and experimental groups. Representative images and quantitative analyses were obtained from anatomically matched sections processed under identical staining conditions.

#### 2.7.2 GFAP immunofluorescence analysis

For assessment of astrocytic activation, immunofluorescence staining for GFAP was performed. Paraffin-embedded brain sections were deparaffinized in xylene and rehydrated through a graded ethanol series. Antigen retrieval was performed in citrate buffer (10mM, pH6.0) for 20 min. After rinsing in PBST, sections were blocked with 5% bovine serum albumin (BSA; Sigma-Aldrich, St. Louis, MO, United States) for 1 h at room temperature to minimize non-specific binding. Sections were subsequently incubated overnight at 4°C with a primary antibody against GFAP (rabbit polyclonal, 1:200 dilution; Genetex, CA, United States), diluted in SignalStain^®^ antibody diluent (Cell Signaling Technology, Massachusetts, United States), to detect astrocyte activation as a marker of pro-inflammatory signaling. Following primary antibody incubation, the sections were washed thrice with PBST and incubated with a goat anti-rabbit IgG Alexa Fluor 555 secondary antibody (1:200 dilution, Invitrogen, MA, United States) for 3 h at room temperature in the dark. Sections were subsequently mounted with EverBrite™ Hardset Mounting Medium (Biotium, California, United States) containing 4′,6-diamidino-2-phenylindole (DAPI).

GFAP immunoreactivity was evaluated in both the striatum and SNpc using anatomically matched coronal sections. In both regions, astrocytic activation was quantified as the density of GFAP-positive cells within the defined ROI. Values from the lesioned side were normalized to the corresponding contralateral side and expressed as percentages. This approach was chosen to account for inter-animal variability in baseline astrocyte distribution and fluorescence intensity.

#### 2.7.3 Ionized calcium-binding adaptor molecule 1 immunofluorescence analysis

To assess microglial activation, immunofluorescence staining for Iba1 was performed on paraffin-embedded brain sections. After deparaffinization, antigen retrieval, and blocking as described above, sections were incubated overnight at 4°C with a primary antibody against Iba1 (rabbit polyclonal, 1:100 dilution, Genetex, California, United States), followed by incubation with a goat anti-rabbit IgG Alexa Fluor 555 secondary antibody (1:200 dilution, Invitrogen, MA, United States). Iba1 immunoreactivity was quantified in the striatum. Iba1-positive cell density was quantified within the lesioned striatum, and expressed as cell density.

#### 2.7.4 Interleukin-6 immunohistochemistry analysis

To assess pro-inflammatory cytokine expression, immunohistochemical staining for IL-6 was performed on paraffin-embedded brain sections. Following deparaffinization, antigen retrieval, and blocking, sections were incubated overnight at 4°C with a rabbit polyclonal anti–IL-6 antibody (1:100 dilution; Genetex, CA, United States). Sections were subsequently incubated with a biotinylated secondary antibody and visualized using the VECTASTAIN^®^ Elite^®^ ABC Kit with DAB substrate. IL-6 expression was quantified as the density of IL-6–positive cells within the lesioned striatum. Values were normalized to the corresponding contralateral side and expressed as percentages. Contralateral normalization was applied to account for inter-animal variability in baseline cytokine expression and staining intensity.

#### 2.7.5 Image acquisition and quantification

Fluorescence and brightfield images were acquired using the same microscope system (BX51, Olympus, Tokyo, Japan) under identical imaging settings for all experimental groups. Image analysis was performed using ImageJ software (version 1.53; National Institutes of Health, Bethesda, MD, United States). Automated thresholding was applied for all quantitative analyses, with identical threshold parameters used within each experiment to minimize operator bias.

The number of animals included in each quantitative analysis varied slightly across markers and brain regions. These differences were attributable to technical factors inherent to histological processing, including tissue integrity, section quality, and regional completeness of the target structures, rather than experimental group allocation or selective exclusion. All available high-quality sections meeting predefined anatomical and technical criteria were included in the corresponding analyses, and exact sample sizes for each outcome measure are reported in the figure legends.

### 2.8 Statistical analysis

Data are presented as mean ± standard error of the mean (SEM). Group differences were evaluated using one-way analysis of variance (ANOVA), followed by Tukey’s honest significant difference *post-hoc* test for pairwise comparisons when appropriate. Comparisons between two groups were performed using Student’s *t*-test. A *p*-value < 0.05 was considered statistically significant. Statistical analyses were performed with GraphPad Prism software (version 8.0.2; GraphPad Software, La Jolla, CA, United States). Detailed statistical methods and sample sizes are provided in the corresponding figure legends.

## 3 Results

### 3.1 FUS induced localized BBB opening without causing histological damage

To determine the optimal FUS output for safe and effective BBB opening, mice were sonicated at 40, 54, or 75mV, corresponding to acoustic pressures of 0.11, 0.15, and 0.28MPa, respectively. BBB permeability was assessed using EB extravasation, and tissue integrity was evaluated through histological analysis.

**FIGURE 2:**
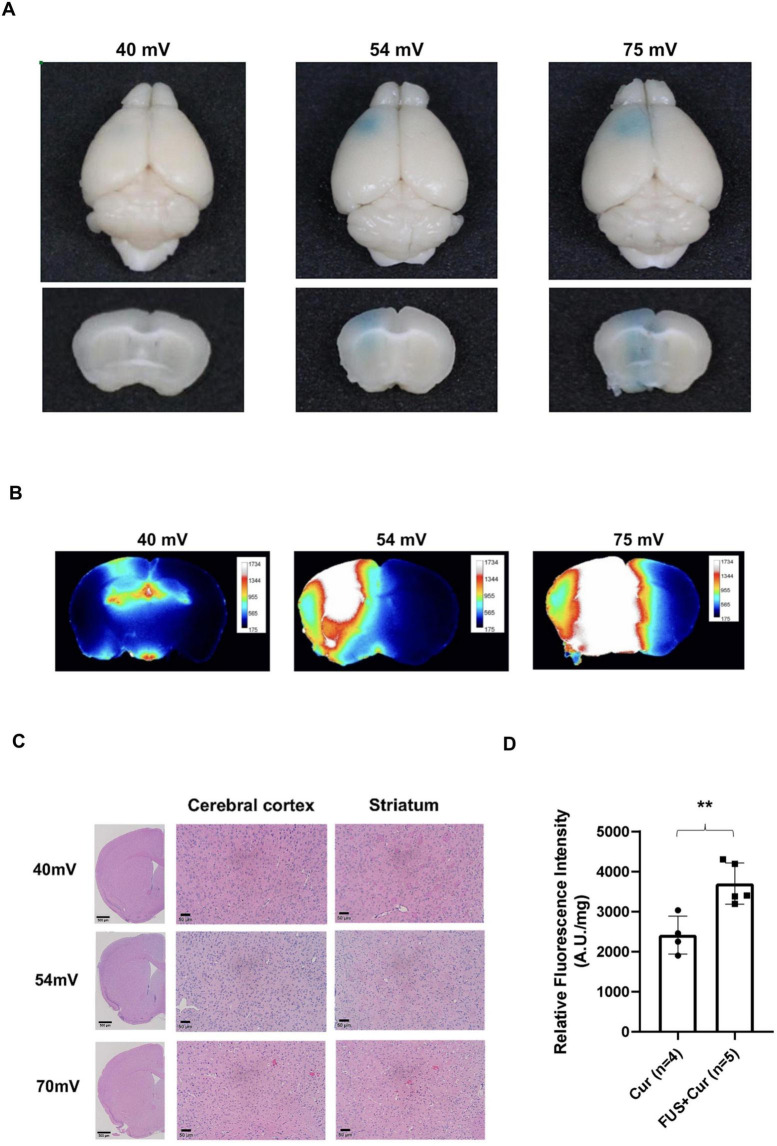
Focused ultrasound (FUS)-induced blood–brain barrier (BBB) opening, histological evaluation, and enhancement of curcumin brain delivery. **(A)** Representative coronal brain sections from mice injected with Evans blue (EB) dye following FUS at 40, 54, or 75 m V (*n* = 3). No visible EB leakage was observed in the 40 mV group. In contrast, localized EB accumulation was detected in the striatum of the 54 mV group, indicating successful and focal BBB opening. The 54 mV group exhibited pronounced EB staining. While no hemorrhagic signs were noted in the 40 and 54 mV groups, visible bleeding was observed in the 75 mV group. **(B)** Macroscopic brain images under each FUS condition. In the 40mV group, sonication did not reach the deep brain structures. In contrast, both the 54 and 75mV groups demonstrated effective targeting of the striatal region. **(C)** Hematoxylin and eosin-stained coronal brain sections of the cerebral cortex and striatum under each FUS condition. Low-magnification images (4 × ) encompassing the sonicated region are shown, together with higher-magnification views (20 × ) to illustrate local tissue morphology. No histological evidence of hemorrhage, edema, or neuronal damage was found in the 40 and 54 mV groups. Mild hemorrhagic changes were observed in the 75mV group. **(D)** Curcumin fluorescence in brain homogenates was measured 3 h after injection. Fluorescence intensity was normalized to total protein concentration and expressed as arbitrary units per milligram of protein (A.U./mg protein). At 3 h post-injection, the FUS + Curcumin group (*n* = 5) showed significantly higher normalized fluorescence intensity compared to the Curcumin-only group (*n* = 4), indicating enhanced delivery via FUS-mediated BBB opening. Statistical significance was determined using Student’s *t*-test. **p < 0.01.

No detectable EB leakage was observed in the 40 mV group, as shown in the coronal sections (Figure 2A) and macroscopic images (Figure 2B), indicating insufficient BBB opening at this acoustic intensity. In the 54 mV group, localized EB accumulation was observed in the sonicated striatum, indicating successful and focal BBB opening without evidence of widespread leakage. In contrast, the 75mV group exhibited pronounced EB staining accompanied by macroscopic evidence of hemorrhage, suggesting tissue damage at higher acoustic intensities.

Figure 2C shows representative H&E-stained brain sections. No evidence of hemorrhage, edema, or neuronal damage were observed in the 40 and 54 mV groups. In contrast, mild hemorrhagic changes were observed in the 75 mV group, demonstrating that while FUS at 40–54 mV safely opens the BBB, higher acoustic intensity may cause tissue injury. Based on the combination of effective EB extravasation and absence of histopathological abnormalities, 54 mV (acoustic pressure = 0.15 MPa) was selected as the optimal FUS parameter for subsequent experiments, enabling localized BBB opening while preserving tissue integrity.

### 3.2 FUS enhances curcumin accumulation in the brain

To evaluate whether FUS-mediated BBB opening enhanced curcumin delivery to the brain, curcumin fluorescence was quantified in brain homogenates 3 h post-injection. Fluorescence intensity was normalized to total protein content and expressed as A.U./mg of protein.

At 3 h post-injection, mice in the FUS + Curcumin group exhibited significantly higher normalized curcumin fluorescence in brain tissue compared with mice receiving curcumin alone (*p* = 0.0063) (Figure 2D), indicating that transient BBB opening induced by FUS enhances intracerebral curcumin accumulation.

### 3.3 Effects of FUS and curcumin on motor and exploratory behaviors

**FIGURE 3:**
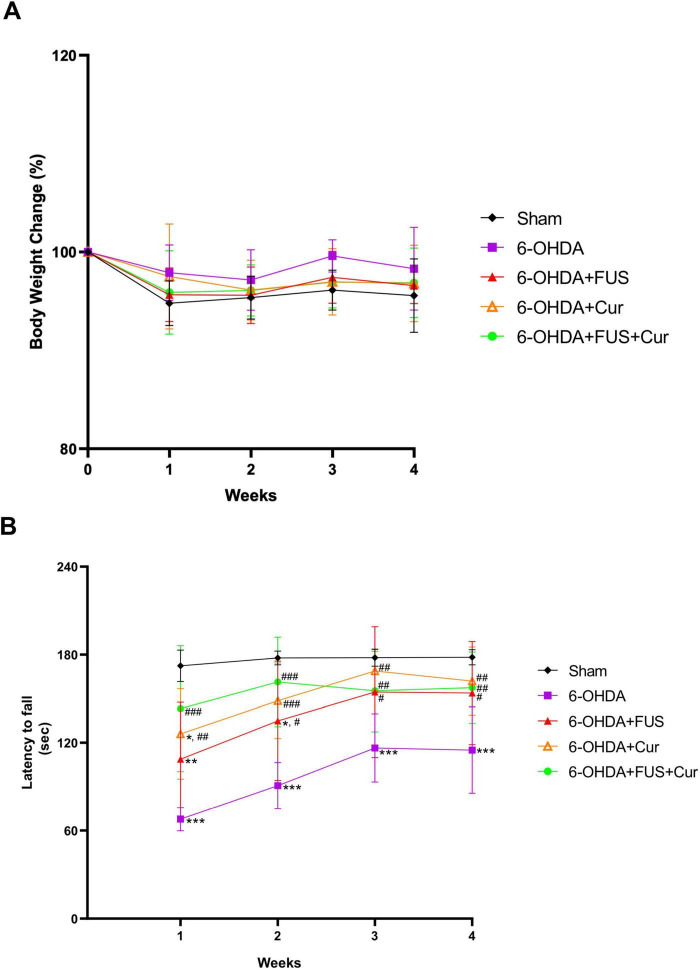
Body weight changes and rotarod performance during the 4-week treatment period. **(A)** Body weight was recorded weekly for all experimental groups throughout the 4-week treatment and observation period. Data are presented as mean ± SEM. No significant differences in body weight were observed among the groups. **(B)** Latency to fall on the rotarod was measured weekly over the 4-week treatment period to assess motor coordination and balance. Data are presented as mean ± SEM. Statistical analysis was performed using one-way ANOVA followed by Tukey’s *post-hoc* test. Symbols indicate the following comparisons: *p and ^#^*p* < 0.05 versus the Sham, 6-OHDA groups, respectively; corresponding double symbols (**p, ^##^*p*) indicate *p* < 0.01, and triple symbols (***p, ^###^*p*) indicate *p* < 0.001 for the same group comparisons, when statistically significant. Group sizes were as follows: Sham (*n* = 8), 6-OHDA (*n* = 9), 6-OHDA + FUS (*n* = 5), 6-OHDA + Curcumin (*n* = 9), and 6-OHDA + FUS + Curcumin (*n* = 9).

Throughout the 4-week treatment and observation period, no significant differences in body weight were detected among the experimental groups (Figure 3A), indicating that the interventions, including repeated FUS and curcumin administration, were well tolerated.

Motor performance was evaluated weekly using the rotarod test (Figure 3B). Across all 4 weeks, the 6-OHDA group exhibited significantly shorter latencies to fall compared with the Sham group (week 1, *p* < 0.0001; week 2, *p* < 0.0001; week 3, *p* = 0.0002; week 4, *p* < 0.0001), confirming persistent motor impairment induced by nigrostriatal lesioning. The 6-OHDA + Curcumin group also exhibited significantly improved rotarod performance compared with the 6-OHDA group at all-time points (week 1, *p* = 0.0018; week 2, *p* = 0.0004; week 3, *p* = 0.0013; week 4, *p* = 0.0033). Similarly, the 6-OHDA + FUS + Curcumin group demonstrated significantly longer latencies to fall compared with the 6-OHDA group throughout the testing period (week 1, *p* < 0.0001; week 2, *p* < 0.0001; week 3, *p* = 0.0251; week 4, *p* = 0.0094).

Direct comparisons between the 6-OHDA + Curcumin and 6-OHDA + FUS + Curcumin groups did not reveal statistically significant differences in latency to fall at any time point. Nevertheless, during the early phase of treatment (weeks 1 and 2), the 6-OHDA + FUS + Curcumin group showed longer latencies to fall compared with the curcumin-only group, whereas rotarod performance in the two groups appeared comparable by weeks 3 and 4. The 6-OHDA + FUS group showed modest improvement compared with the 6-OHDA group at weeks 2–4 (week 2, *p* = 0.0114; week 3, *p* = 0.0376; week 4, *p* = 0.0264), although its performance did not differ significantly from that of the curcumin-treated groups. Overall, these results indicate that curcumin treatment is associated with improved motor coordination in the rotarod test, while the addition of FUS does not further increase overall rotarod performance but may be associated with earlier improvement during the initial treatment phase.

**FIGURE 4:**
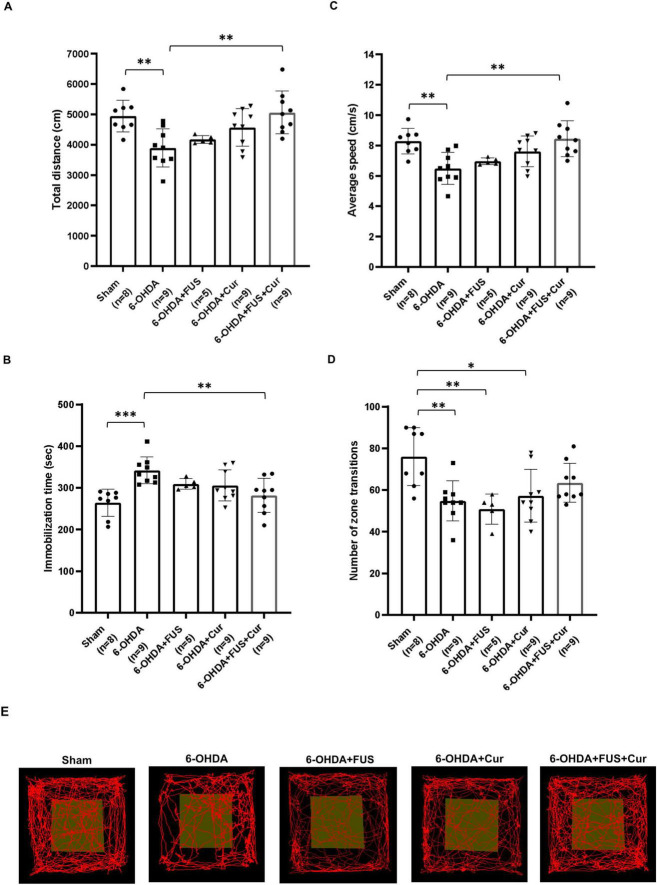
Open field test evaluating locomotor activity and exploratory behavior after 4 weeks of treatment. Open field performance was assessed by measuring **(A)** total distance traveled, **(B)** immobilization time, **(C)** average speed and **(D)** the number of zone transitions, with **(E)** showing representative movement paths for each group. Data are presented as mean ± SEM. The dots represent individual mice in each group (Sham group, *n* = 8; 6-OHDA group, *n* = 9; 6-OHDA + FUS group, *n* = 5; 6-OHDA + Curcumin group, *n* = 9; and 6-OHDA + FUS + Curcumin group, *n* = 9). Significant differences were determined using one-way ANOVA followed by Tukey’s *post-hoc* test and are indicated with asterisks. *p < 0.05, **p < 0.01, ***p < 0.001.

Spontaneous locomotor activity and exploratory behavior were assessed using the open field test after 4 weeks of treatment (Figure 4). Compared with the Sham group, the 6-OHDA group exhibited significant reductions in total distance traveled (*p* = 0.0068), average velocity (*p* = 0.0051), and zone transitions (*p* = 0.0033), together with increased immobilization time (*p* = 0.0004), indicating marked deficits in locomotion and exploration. The 6-OHDA + FUS + Curcumin group showed significant improvements relative to the 6-OHDA group in total distance traveled (*p* = 0.0015), average velocity (*p* = 0.0014), and immobilization time (*p* = 0.0058). In contrast, the 6-OHDA + Curcumin and 6-OHDA + FUS groups did not exhibit significant improvements compared with the 6-OHDA group across these open field parameters. Direct comparisons between the 6-OHDA + Curcumin and 6-OHDA + FUS + Curcumin groups did not reveal statistically significant differences in total distance, velocity, immobilization time, or zone transitions. In addition, FUS alone did not produce significant behavioral improvement in the absence of curcumin. Taken together, these findings indicate that curcumin treatment is associated with improved motor coordination in the rotarod test, whereas the combination of FUS and curcumin is associated with more consistent improvement in spontaneous locomotor activity in the open field test.

### 3.4 Effects of FUS and curcumin on striatal dopaminergic fibers

**FIGURE 5:**
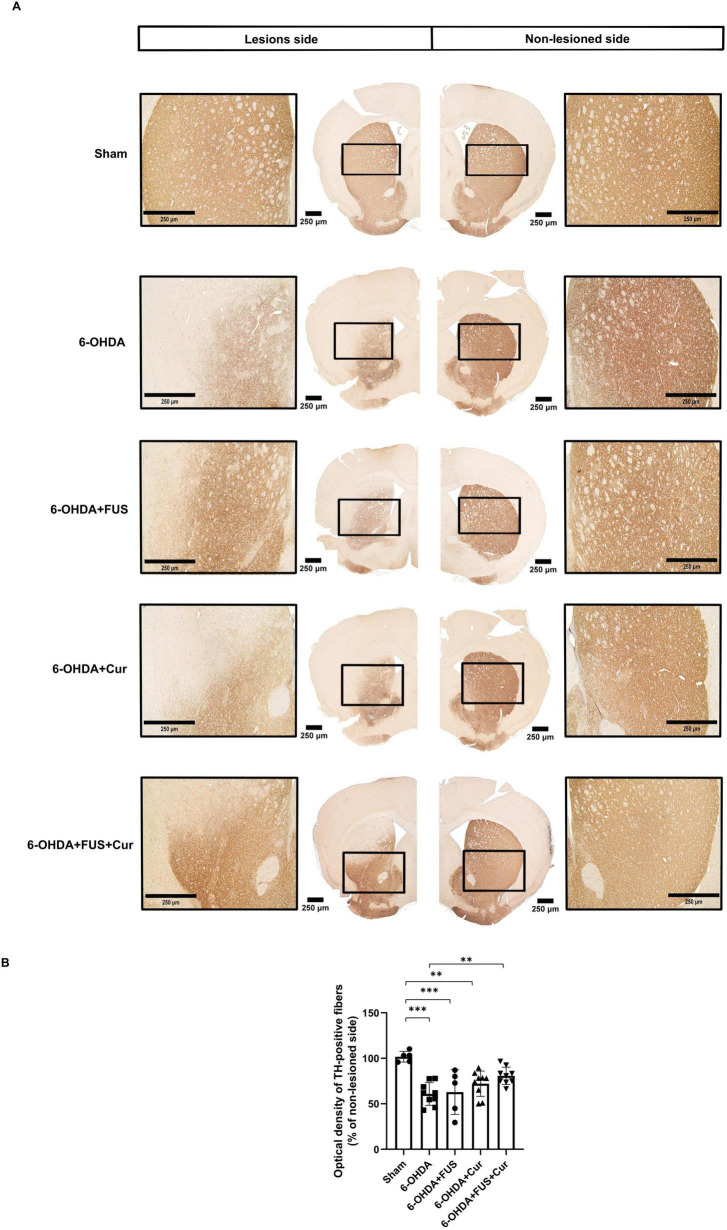
Tyrosine hydroxylase (TH) immunohistochemistry evaluating dopaminergic fiber integrity in the striatum after 4 weeks of treatment. **(A)** Representative TH-immunostained coronal sections of the striatum from each experimental group, obtained from anatomically matched anteroposterior levels corresponding to the central striatal region and processed simultaneously under identical staining conditions. Low-magnification images were acquired at 4 × magnification, with representative higher-magnification images (20 × ) shown to illustrate local differences in TH-positive fiber density. The 6-OHDA group showed marked loss of TH-positive fibers compared with the Sham group. Visual preservation of TH immunoreactivity was observed in the 6-OHDA + FUS + Curcumin group relative to the 6-OHDA group. **(B)** Quantification of striatal TH immunoreactivity, expressed as optical density of TH-positive fibers in the lesioned striatum and normalized to the contralateral side. Data are presented as mean ± SEM. The dots represent individual mice in each group (Sham group, *n* = 5; 6-OHDA group, *n* = 8; 6-OHDA + FUS group, *n* = 5; 6-OHDA + Curcumin group, *n* = 7; and 6-OHDA + FUS + Curcumin group, *n* = 9). Significant differences were determined using one-way ANOVA followed by Tukey’s *post-hoc* test and are indicated with asterisks. **p < 0.01, ***p < 0.001.

To evaluate the effects of curcumin treatment and FUS-mediated delivery on nigrostriatal dopaminergic fibers, TH immunohistochemistry was performed to assess dopaminergic neuron integrity in the lesioned striatum (Figure 5A). The 6-OHDA-lesioned group showed a marked reduction in TH-positive staining compared to the Sham group, indicating significant dopaminergic neuron loss induced by the neurotoxin. Notably, the 6-OHDA + FUS + Curcumin group demonstrated greater preservation of TH-positive fibers relative to the 6-OHDA group.

These observations were supported by quantitative analysis of striatal TH immunoreactivity, expressed as optical density normalized to the contralateral side (Figure 5B). The 6-OHDA group exhibited a significant reduction in TH optical density compared with the Sham group (*p* < 0.0001). TH immunoreactivity in the 6-OHDA + FUS group remained significantly lower than that in the Sham group (*p* = 0.0008) and did not differ from the 6-OHDA group (*p* = 0.9987), indicating that FUS alone did not preserve striatal dopaminergic fibers. Similarly, the 6-OHDA + Curcumin group showed reduced TH optical density compared with the Sham group (*p* = 0.0043), with no significant difference relative to the 6-OHDA group (*p* = 0.4261).

In contrast, the 6-OHDA + FUS + Curcumin group showed significantly higher striatal TH optical density compared with the 6-OHDA group (*p* = 0.0030), whereas its TH immunoreactivity did not differ significantly from that of the Sham group (*p* = 0.0686). Direct comparisons among treatment groups revealed no significant differences between the 6-OHDA + Curcumin and 6-OHDA + FUS + Curcumin groups (*p* = 0.6751), nor between the 6-OHDA + FUS and curcumin-treated groups. These results indicate that increased striatal TH preservation was observed only in the group receiving combined FUS and curcumin treatment.

### 3.5 Effects of FUS and curcumin on dopaminergic neurons in the SNpc

**FIGURE 6:**
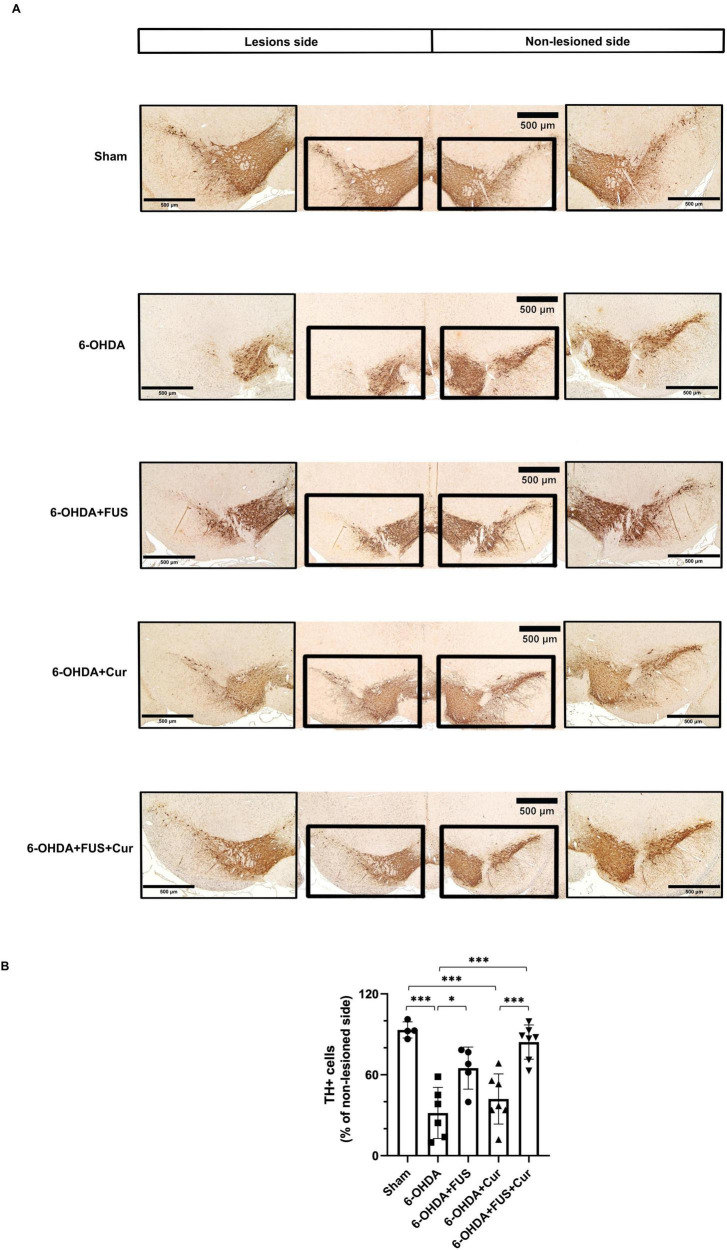
Tyrosine hydroxylase (TH) immunohistochemistry evaluating dopaminergic neuron preservation in the substantia nigra pars compacta (SNpc) after 4 weeks of treatment. **(A)** Representative TH-immunostained coronal sections of the substantia nigra pars compacta (SNpc) from each experimental group, obtained from anatomically matched anteroposterior levels and processed simultaneously under identical staining conditions. Low-magnification images were acquired at 4 × magnification. Higher-magnification images (10 × ) are shown for comparison of TH-positive dopaminergic neurons. The 6-OHDA group exhibited a marked reduction in TH-positive neurons in the SNpc. Increased TH immunoreactivity was observed in the treatment groups, with the 6-OHDA + FUS + Curcumin group showing greater preservation of TH-positive neurons relative to the 6-OHDA group. **(B)** Quantification of TH-positive cells in the SNpc, expressed as the number of TH-immunoreactive neurons in the lesioned SNpc and normalized to the contralateral side. Data are presented as mean ± SEM. Dots represent individual animals in each group (Sham group, *n* = 4; 6-OHDA group, *n* = 6; 6-OHDA + FUS group, *n* = 5; 6-OHDA + Curcumin group, *n* = 7; 6-OHDA + FUS + Curcumin group, *n* = 7). Statistical significance was determined using one-way ANOVA followed by Tukey’s *post-hoc* test and is indicated with asterisks. *p < 0.05, ***p < 0.001.

To further evaluate the effects of FUS and curcumin on the nigrostriatal pathway, TH immunohistochemistry was performed to assess dopaminergic neuron survival in the SNpc (Figure 6A). The 6-OHDA-lesioned group exhibited a pronounced reduction in TH-positive neurons compared with the Sham group, confirming substantial dopaminergic neuronal loss induced by the neurotoxin.

Quantitative analysis of TH-positive cell counts in the SNpc, normalized to the contralateral side, revealed distinct treatment-related effects (Figure 6B). Compared with the 6-OHDA group, the 6-OHDA + FUS + Curcumin group showed a marked increase in TH-positive neuron density (*p* < 0.0001), whereas no significant difference was observed between the 6-OHDA and 6-OHDA + Curcumin groups (*p* = 0.7616). Notably, direct comparison between the 6-OHDA + Curcumin and 6-OHDA + FUS + Curcumin groups demonstrated a significant difference in favor of the combined treatment (*p* = 0.0003), indicating enhanced preservation of dopaminergic neurons in the SNpc when curcumin delivery was combined with FUS.

The 6-OHDA + FUS group also exhibited a modest but statistically significant increase in TH-positive neuron density compared with the 6-OHDA group (*p* = 0.0145). However, this group did not differ significantly from the 6-OHDA + Curcumin group (*p* = 0.1253) or the 6-OHDA + FUS + Curcumin group (*p* = 0.2544), indicating that the effects of FUS alone on TH-positive neuron density in the SNpc were modest compared with those observed with combined treatment.

When compared with the Sham group, TH-positive neuron density in the 6-OHDA + Curcumin group remained significantly reduced (*p* = 0.0002), whereas no significant difference was observed between the Sham and 6-OHDA + FUS + Curcumin groups (*p* = 0.8895). These findings indicate that combined FUS and curcumin treatment was associated with marked preservation of dopaminergic neurons in the SNpc, such that TH-positive cell counts did not differ significantly from those observed in the Sham group.

### 3.6 FUS-enhanced curcumin delivery attenuates astrogliosis in the striatum

**FIGURE 7:**
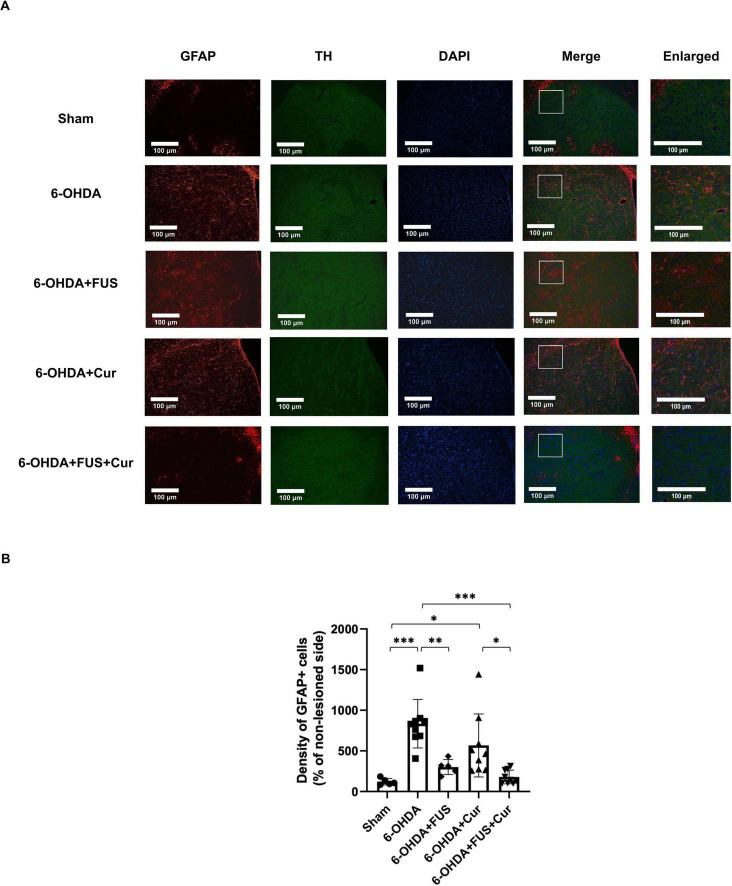
Glial fibrillary acidic protein (GFAP) immunofluorescence analysis of astrocytic activation in the striatum after 4 weeks of treatment. **(A)** Representative GFAP immunofluorescence images showing astrocytic activation in the lesioned striatum from each experimental group. Images were obtained from anatomically matched coronal sections and acquired using identical imaging parameters at 20 × magnification. Representative higher-magnification images (40 × ) are shown to illustrate regional differences in GFAP expression. The 6-OHDA-lesioned group exhibited increased GFAP immunoreactivity, indicative of reactive astrogliosis. **(B)** Quantification of GFAP-positive cell density within regions of interest encompassing the striatum, normalized to the contralateral side and expressed as a percentage. Data are presented as mean ± SEM. The dots represent individual mice in each group (Sham group, *n* = 5; 6-OHDA group, *n* = 9; 6-OHDA + FUS group, *n* = 5; 6-OHDA + Curcumin group, *n* = 9; and 6-OHDA + FUS + Curcumin group, *n* = 9). Significant differences were determined using one-way ANOVA followed by Tukey’s *post-hoc* test and are indicated with asterisks. *p < 0.05, **p < 0.01, ***p < 0.001.

To assess astrogliosis following 6-OHDA–induced neurotoxicity and the effects of FUS-mediated curcumin delivery, GFAP immunofluorescence staining was performed in the lesioned striatum (Figure 7A). Compared with the Sham group, the 6-OHDA-lesioned group exhibited a marked increase in GFAP-positive astrocytes, indicating pronounced reactive astrogliosis.

These observations were corroborated by quantitative analysis of GFAP-positive cell density (Figure 7B). The 6-OHDA + FUS group showed a modest but significant reduction in GFAP-positive cell density compared with the 6-OHDA group (*p* = 0.0050), indicating that FUS alone may influence astrocytic responses in the lesioned striatum. Curcumin treatment alone did not result in a statistically significant reduction in GFAP-positive cell density relative to the 6-OHDA group (*p* = 0.1839). In contrast, the 6-OHDA + FUS + Curcumin group exhibited the lowest GFAP-positive cell density among all lesioned groups and showed a significant reduction compared with the 6-OHDA group (*p* < 0.0001). Moreover, GFAP-positive cell density in the 6-OHDA + FUS + Curcumin group was significantly lower than that in the 6-OHDA + Curcumin group (*p* = 0.0193), whereas no significant difference was observed between the 6-OHDA + FUS and 6-OHDA + FUS + Curcumin groups (*p* = 0.9034). Together, these findings indicate that the combination of FUS and curcumin was associated with a greater attenuation of striatal astrogliosis than curcumin treatment alone, while FUS alone exerted a limited but detectable effect on astrocytic activation.

### 3.7 FUS-enhanced curcumin delivery attenuates astrogliosis in the SNPc

**FIGURE 8:**
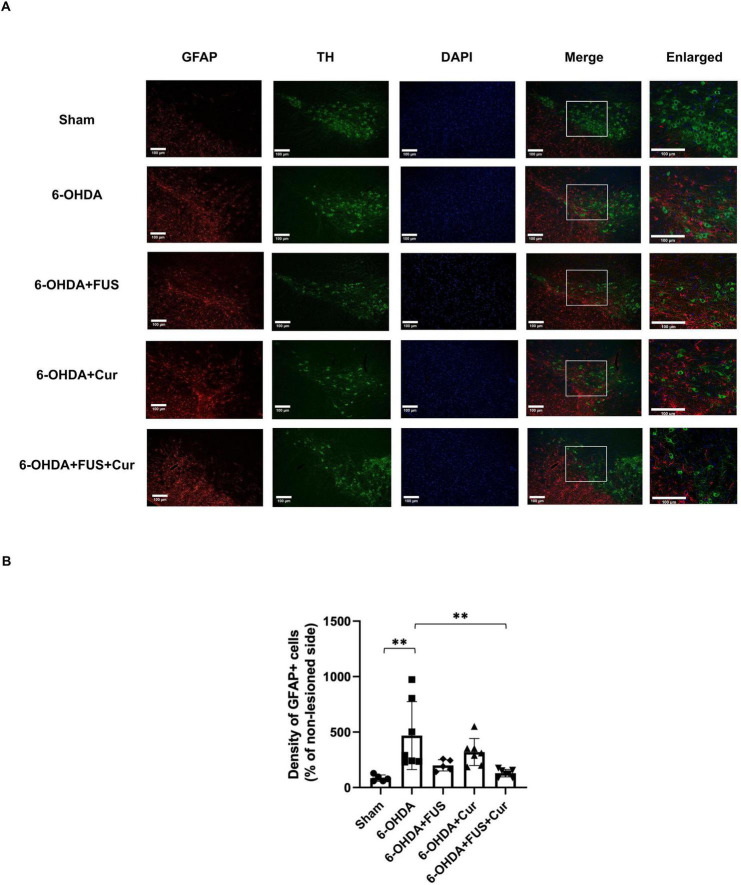
Glial fibrillary acidic protein (GFAP) immunofluorescence analysis of astrocytic activation in the substantia nigra pars compacta (SNpc) after 4 weeks of treatment. **(A)** Representative GFAP immunofluorescence images showing astrocytic activation in the lesioned SNpc from each experimental group. Images were obtained from anatomically matched coronal sections and acquired using identical imaging parameters at 20 × magnification. Representative higher-magnification images (40 × ) are shown to illustrate regional differences in GFAP expression. The 6-OHDA-lesioned group exhibited increased GFAP immunoreactivity, consistent with astrogliosis. **(B)** Quantification of GFAP-positive cell density within regions of interest encompassing the SNpc, normalized to the contralateral side and expressed as a percentage. Data are presented as mean ± SEM. Dots represent individual animals in each group (Sham group, *n* = 5; 6-OHDA group, *n* = 7; 6-OHDA + FUS group, *n* = 5; 6-OHDA + Curcumin group, *n* = 7; 6-OHDA + FUS + Curcumin group, *n* = 7). Statistical significance was determined using one-way ANOVA followed by Tukey’s *post-hoc* test and is indicated with asterisks. **p < 0.01.

To further determine whether the modulatory effects of FUS-mediated curcumin delivery on astrocytic activation extended to the nigrostriatal cell body region, GFAP immunofluorescence staining was performed in the SNpc (Figure 8A).

Quantitative analysis of GFAP-positive cell density normalized to the contralateral side is shown in Figure 8B. Compared with the Sham group, the 6-OHDA-lesioned group exhibited a significant increase in GFAP-positive astrocytes in the SNpc (*p* = 0.0033), consistent with astrocytic activation following dopaminergic neuron degeneration. The 6-OHDA + FUS group did not differ significantly from the 6-OHDA group (*p* = 0.0599), indicating that FUS alone did not substantially modify astrocytic activation in the SNpc. Similarly, curcumin treatment alone did not result in a significant reduction in GFAP-positive cell density compared with the 6-OHDA group (*p* = 0.4384).

In contrast, the 6-OHDA + FUS + Curcumin group showed a significant reduction in GFAP-positive cell density relative to the 6-OHDA group (*p* = 0.0046). Direct comparisons between the 6-OHDA + FUS + Curcumin group and the 6-OHDA + Curcumin group did not reach statistical significance (*p* = 0.2059), nor was a difference observed between the 6-OHDA + FUS and 6-OHDA + FUS + Curcumin groups (*p* = 0.9425). Overall, reduced astrocytic activation in the SNpc was observed primarily in animals receiving combined FUS and curcumin treatment, whereas neither FUS nor curcumin alone was associated with statistically significant changes at this anatomical level.

### 3.8 Effects of FUS and curcumin on microglial activation and pro-inflammatory cytokine expression in the striatum

**FIGURE 9:**
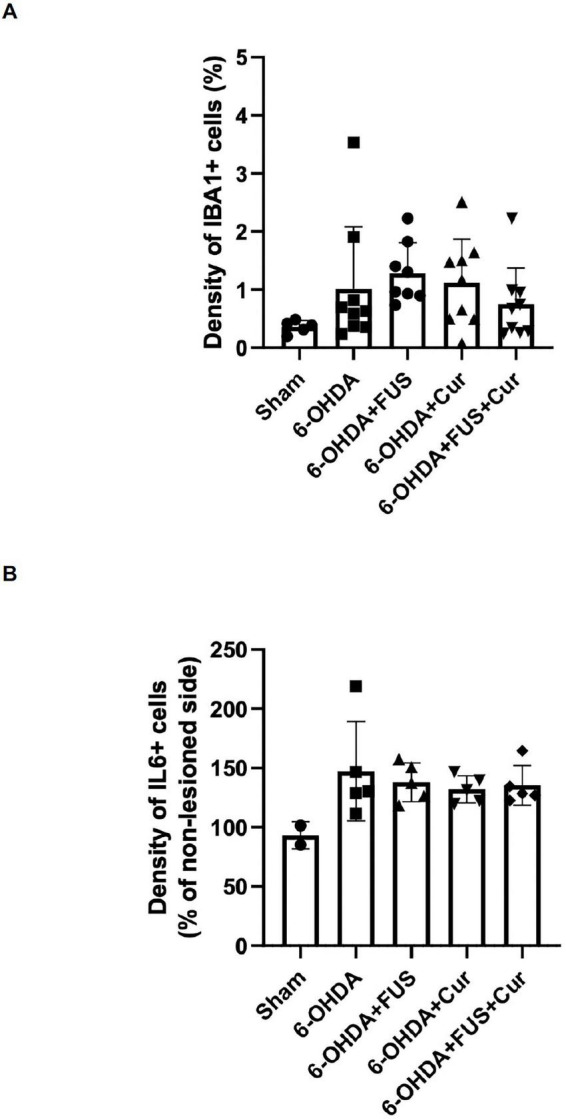
Microglial activation and pro-inflammatory cytokine expression in the striatum after 4 weeks of treatment. **(A)** Quantification of Iba1-positive cell density in the lesioned striatum. Iba1 immunofluorescence was analyzed in anatomically matched coronal sections, and signal density within the striatal region of interest was quantified and expressed as mean ± SEM. Dots represent individual animals in each group (Sham group, *n* = 5; 6-OHDA group, *n* = 9; 6-OHDA + FUS group, *n* = 8; 6-OHDA + Curcumin group, *n* = 9; 6-OHDA + FUS + Curcumin group, *n* = 9). Statistical significance was determined using one-way ANOVA followed by Tukey’s *post-hoc* test. **(B)** Quantification of IL-6 immunoreactivity in the lesioned striatum. IL-6-positive cell density was quantified within the striatal region of interest, normalized to the contralateral side, and expressed as mean ± SEM. Dots represent individual animals in each group (Sham group, *n* = 2; 6-OHDA group, *n* = 5; 6-OHDA + FUS group, *n* = 5; 6-OHDA + Curcumin group, *n* = 5; 6-OHDA + FUS + Curcumin group, *n* = 5). Statistical significance was determined using one-way ANOVA followed by Tukey’s *post-hoc* test.

To further examine whether FUS-mediated curcumin delivery modulated microglial activation and inflammatory signaling in the lesioned striatum, immunofluorescence staining for Iba1 and immunohistochemical staining for IL-6 were performed (Figure 9).

Quantitative analysis of Iba1-positive cell density in the lesioned striatum is shown in Figure 9A. No significant differences were observed between the Sham and 6-OHDA groups (*p* = 0.5026), nor were differences detected among the 6-OHDA, 6-OHDA + FUS, 6-OHDA + Curcumin, and 6-OHDA + FUS + Curcumin groups. Under the present experimental conditions, striatal microglial density, as assessed by Iba1 immunoreactivity, did not differ significantly among groups treated with FUS, curcumin, or their combination.

Assessment of pro-inflammatory cytokine expression was performed by quantifying IL-6–positive cell density in the lesioned striatum (Figure 9B). IL-6 immunoreactivity did not differ significantly between the Sham and 6-OHDA groups (*p* = 0.1004), and no significant differences were observed among the 6-OHDA, 6-OHDA + FUS, 6-OHDA + Curcumin, and 6-OHDA + FUS + Curcumin groups. Comparisons between treatment groups also did not reach statistical significance. Collectively, in contrast to the marked changes observed in astrocytic activation, microglial density and IL-6 immunoreactivity in the striatum remained relatively unchanged across experimental groups at the 4-week time point.

## 4 Discussion

This study demonstrates that repeated FUS at 0.15 MPa safely and reversibly opens the BBB in the striatum of 6-OHDA lesioned mice, thereby enhancing the brain delivery of native curcumin and improving behavioral and histological outcomes. Four weekly FUS sessions combined with curcumin administration produce significant improvements in motor function, as assessed through rotarod and open field tests, and were associated with preservation of the nigrostriatal pathway, including increased TH immunoreactivity in the lesioned striatum and improved TH-positive dopaminergic neuron survival in the SNpc. In addition, combined treatment attenuated astrocytic activation in both the striatum and SNpc. These findings support the notion that FUS-facilitated BBB opening enhances the therapeutic efficacy of curcumin in PD. Unlike previous studies that employ nanoparticles to improve delivery (Lei et al., 2023; Yan et al., 2021; Zhang et al., 2018), this study is the first to investigate unmodified curcumin combined with FUS in a PD mouse model, demonstrating that this approach is associated with meaningful functional and histological improvements. This strategy offers a less complex and potentially more accessible alternative for clinical translation without requiring additional carrier systems.

The strengths of this study include repeated FUS sessions, targeted BBB opening in the striatum, and comprehensive safety evaluation. We implemented four FUS sessions over 4 weeks, demonstrating that repeated treatments are well tolerated, with no evidence of edema or hemorrhage. These findings align with the safety profiles reported in preclinical and early phase human trials of FUS-mediated BBB opening in PD (Gasca-Salas et al., 2021; Ji et al., 2019; Lin et al., 2020; Wang et al., 2022), supporting the feasibility of sustained therapeutic regimens in this population.

Furthermore, effective and focal BBB opening occurs at a relatively low peak negative pressure of 0.15 MPa in this study, which is markedly lower than the 0.24–0.45 MPa used in the nanobubble-mediated curcumin delivery protocol by Yan et al. (2021), or the 0.45 MPa applied by Ji et al. for FUS-enhanced neurotrophin delivery (Ji et al., 2019). Several methodological factors likely contribute to this enhanced efficiency. First, sonication targets a small, localized region of the mouse striatum using a focused transducer with precise stereotaxic alignment, potentially enhancing spatial accuracy and acoustic energy concentration. Second, curcumin was administered immediately after sonication, during the brief window of BBB permeability, which may optimize brain uptake despite the limited solubility of curcumin. Finally, rather than relying on nanoparticle carriers with delayed release profiles, the use of unmodified curcumin may facilitate more rapid diffusion across the transiently permeabilized BBB. This effect likely results from the small molecular size and lipophilic nature of curcumin (Moldoveanu et al., 2024), which enable passive diffusion once the tight junctions are temporarily disrupted by FUS.

This study is the first to employ the 6-OHDA model to assess the therapeutic effects of FUS-mediated curcumin delivery. The 6-OHDA lesion model was selected for its ability to produce focal dopaminergic degeneration along the nigrostriatal pathway, closely resembling the regional pathology observed in PD (Slézia et al., 2023). Direct striatal injection of 6-OHDA enables precise targeting and produces gradual retrograde degeneration over 1–3 weeks (Jagmag et al., 2015), providing a window to test region-specific interventions, including FUS-mediated BBB opening.

The timing of curcumin administration in this study was chosen to address an early-to-intermediate stage of 6-OHDA–induced pathology. Previous studies have shown that prophylactic or very early curcumin treatment, initiated before or immediately after toxin exposure, is associated with reduced oxidative stress, iron accumulation, and early neuroinflammatory activation, together with greater preservation of nigrostriatal dopaminergic neurons (Du et al., 2012; Song et al., 2016; Tripanichkul and Jaroensuppaperch, 2013). Time-course analyses in both rat and mouse 6-OHDA models further indicate that dopaminergic terminal loss in the striatum occurs within hours after toxin injection, whereas dopaminergic neuron loss in the substantia nigra and more pronounced glial responses develop gradually over several days and become evident approximately 6–14 days post-lesion (Stott and Barker, 2014; Walsh et al., 2011). In this study, initiating treatment at 7 days post-lesion allows evaluation of therapeutic effects during an active phase of ongoing neurodegeneration and glial activation, rather than during the acute neurotoxic period. From this perspective, the present study complements prior preventive or early intervention studies by examining the effects of curcumin under conditions in which pathological processes are already established but continue to evolve. In addition, FUS–mediated BBB opening may influence treatment efficacy by enhancing regional drug delivery and by inducing secondary biological effects, including modulation of neuroinflammatory signaling and alterations in the neurovascular microenvironment (Gorick et al., 2022).

While the 1-methyl-4-phenyl-1,2,3,6-tetrahydropyridine (MPTP) model is frequently employ in FUS research owing to its systemic dopaminergic toxicity (Lin et al., 2020; Slézia et al., 2023; Wang et al., 2022; Yan et al., 2021), it does not produce the focal striatal degeneration characteristic of human PD (Jagmag et al., 2015). In mice, MPTP crosses the BBB and is metabolized by glial cells into 1-methyl-4-phenylpyridinium, which selectively enters dopaminergic neurons and inhibits mitochondrial complex I, resulting in widespread and rapidly develop lesions in the substantia nigra and striatum (Jackson-Lewis and Przedborski, 2007). The MPTP model replicates key PD features and is widely employ owing to its reproducibility and ease of systemic administration. However, in mice, it produces more acute and uniform lesions and often requires repeated dosing to maintain effects, differing from the gradual progression observed in human PD (Jagmag et al., 2015).

Given the focal and gradual nature of 6-OHDA-induced lesions (Jagmag et al., 2015), this model is better suited for evaluating localized therapeutic strategies. By selectively disrupting the BBB in the lesioned striatum during curcumin therapy, the study demonstrates region-specific restoration of TH expression and attenuation of glial activation. This spatially targeted therapeutic response indicates that FUS can enhance delivery to specific neural circuits, a critical feature for clinical applications requiring precise localization.

In this study, FUS-facilitated curcumin delivery produces significant improvements in rotarod and open field performance in 6-OHDA-lesioned mice, indicating benefits in motor coordination, balance, spontaneous locomotion, and exploratory behavior (Sheta et al., 2024). Notably, while overall rotarod performance did not differ significantly between the curcumin-only and FUS + curcumin groups across the full 4-week period, the FUS + curcumin group exhibited a trend toward longer latencies to fall during the early treatment phase (weeks 1–2). This temporal pattern suggests that focal BBB opening may be associated with earlier functional improvement, potentially through increased curcumin availability in the lesioned striatum during a period when dopaminergic terminals are undergoing active degeneration (Lee et al., 1996; Sun et al., 2022). By contrast, assessment of spontaneous locomotor activity using the open field test after 4 weeks of treatment showed differences among experimental groups, with significant improvements observed only in the FUS + curcumin group, whereas curcumin or FUS administered alone was not associated with statistically significant changes. The patterns observed between the rotarod and open field assessments may relate to the fact that these behavioral measures emphasize different components of motor function, with spontaneous locomotor activity being more sensitive to changes in baseline movement and exploratory behavior (Sheta et al., 2024). Enhanced regional delivery of curcumin through FUS-mediated BBB opening may be associated with its antioxidative and anti-inflammatory actions, including modulation of astrocytic activation, attenuation of oxidative stress, and maintenance of mitochondrial function, which together may be linked to improvements in spontaneous motor activity during nigrostriatal degeneration (Nebrisi, 2021). These effects may be associated with better preservation of neuronal function and dopaminergic signaling in surviving neurons during a vulnerable phase of degeneration, suggesting that spatially targeted interventions may help sustain motor performance during the early stages of Parkinsonian pathology.

The nigrostriatal histological findings address an important issue regarding the added contribution of FUS beyond curcumin treatment alone. In the striatum, TH fiber optical density was significantly higher in the FUS + curcumin group compared with the 6-OHDA group, whereas neither curcumin alone nor FUS alone differed significantly from the 6-OHDA condition. At the level of the SNpc, TH-positive dopaminergic neuron counts were significantly higher in the FUS + curcumin group than in the curcumin-only group, indicating that the combination of BBB opening with curcumin delivery was associated with greater preservation of dopaminergic neurons in the cell body region. This regional pattern is consistent with the temporal evolution of 6-OHDA pathology, in which dopaminergic terminal loss in the striatum occurs early, while degeneration of SNpc neurons develops progressively over subsequent days to weeks (Stott and Barker, 2014; Walsh et al., 2011). Under these conditions, enhanced drug access to the lesioned circuit may be associated with a more readily detectable effect on dopaminergic neuron survival in the SNpc than on striatal terminals when treatment is initiated 1 week after lesioning.

The inclusion of a 6-OHDA + FUS group enables comparison of treatment effects in the absence of curcumin. FUS alone was associated with modest changes across several outcomes, including small improvements in rotarod performance at later time points, a statistically significant increase in SNpc TH-positive neuron counts relative to the 6-OHDA group, and reduced astrocytic activation in the striatum. However, FUS alone did not preserve striatal TH fibers and was not associated with significant improvement in spontaneous locomotor activity in the open field assessment. Under the present experimental conditions, BBB opening in the absence of curcumin was not sufficient to yield consistent behavioral improvement or to reproduce the pattern observed with combined treatment across behavioral and histological measures. At the same time, the reductions in striatal astrocytic activation and the modest changes in SNpc dopaminergic neuron counts observed following FUS alone are compatible with prior observations suggesting that ultrasound-mediated BBB opening may influence the neurovascular microenvironment and neuroinflammatory signaling pathway (Gorick et al., 2022).

The therapeutic effects of curcumin observed in this study are consistent with its established role in modulating neuroinflammation, a central pathological process in PD (Garodia et al., 2023; Nebrisi, 2021). Extensive evidence reveals that curcumin inhibits key inflammatory signaling cascades, including suppression of NF-κB activation and downregulation of proinflammatory cytokines such as TNF-α, IL-1β, and IL-6 (Liu et al., 2025). Additionally, it reduces the expression of iNOS and cycloxygenase-2, thereby limiting the production of nitric oxide and prostaglandin, which contribute to oxidative stress and neuronal damage (Nebrisi, 2021). However, the extent and cellular specificity of these effects may depend on disease stage, regional pathology, and treatment timing.

In the present study, neuroinflammatory modulation was most evident at the level of astrocytic activation. FUS-assisted curcumin delivery was associated with a significant reduction in GFAP immunoreactivity in the lesioned striatum and SNpc, whereas curcumin or FUS alone showed more limited or region-specific effects. In contrast, microglial density assessed by Iba1 immunoreactivity and IL-6 expression in the striatum did not differ significantly among experimental groups at the 4-week time point. This pattern suggests that, under the current experimental conditions, astrocytic responses may be more sensitive to curcumin delivery enhanced by FUS than microglial activation or IL-6–related inflammatory signaling.

Astrocytes are increasingly recognized as key regulators of the neuroinflammatory milieu in PD, contributing to oxidative stress, excitotoxicity, and altered metabolic support for dopaminergic neurons (Sonninen et al., 2020). Modulation of astrocytic activation may therefore influence neuronal vulnerability even in the absence of detectable changes in microglial density or selected cytokine markers. Curcumin has been shown to interfere with astrocyte-derived inflammatory signaling and glial crosstalk, which may contribute to modulation of local inflammatory conditions associated with neuronal stress (Nebrisi, 2021; Yu et al., 2016). By attenuating astrocytic reactivity, curcumin helps maintain extracellular homeostasis, reduces oxidative burden, and preserves neuronal function (Yu et al., 2016). In this study, these anti-inflammatory effects likely contribute to the improved motor performance and preservation of TH-positive fibers observed in groups receiving curcumin combined with FUS-mediated delivery. When administered during the early degenerative phase in the 6-OHDA model, curcumin may interrupt the progression of neuroinflammatory and neurodegenerative changes.

The absence of significant changes in Iba1 and IL-6 measures does not exclude broader immunomodulatory effects but may be related to the timing of tissue collection, the focal nature of the 6-OHDA lesion, or the specific inflammatory markers examined. In toxin-based models, microglial activation and cytokine expression can vary across stages of degeneration and may be more prominent earlier in the disease course, whereas astrocytic responses may persist during later phases (Acarin et al., 2000; Bouvier et al., 2022; Gao et al., 2013; Joers et al., 2017). When treatment is initiated during an early-to-intermediate stage of nigrostriatal degeneration, FUS-enhanced curcumin delivery may be more closely associated with changes in astrocytic activation, which could be relevant to local inflammatory regulation and neuronal function.

Although the present findings confirm that FUS effectively enhances intracerebral curcumin delivery, several behavioral and histological outcomes did not differ significantly between the curcumin-only and FUS + curcumin groups. This finding suggests that, under the dosing regimen and treatment duration applied in this study, systemic curcumin administration alone was sufficient to induce measurable functional and histological effects, which may have limited the ability to detect additional benefits attributable to enhanced delivery. In addition, inherent interindividual variability in behavioral performance and histological measures, together with modest group sizes and a relatively short observation period, may have reduced sensitivity for identifying smaller between-group differences. Nevertheless, the FUS + curcumin treatment group showed significantly greater preservation of TH-positive dopaminergic neurons in the SNpc and a greater reduction in striatal GFAP-positive astrocyte density compared with curcumin treatment alone, supporting an added contribution of FUS-assisted delivery on selected nigrostriatal and astroglial measures, even when behavioral measures did not differ significantly between the two curcumin-treated groups. Future studies using lower curcumin doses, longer treatment durations, or incorporating pharmacokinetic analyses could help clarify the dose–response relationship and strengthen the translational potential of FUS-mediated curcumin delivery.

Compared with nanoparticle-based delivery systems, the use of unmodified curcumin combined with FUS offers several unique advantages. First, it eliminates the need for synthetic carriers, thereby reducing potential concerns related to nanoparticle biocompatibility, long-term retention, or unforeseen immunogenic or inflammatory reactions in the brain (Fortune et al., 2025; Patra et al., 2018). Second, FUS-mediated BBB opening enables transient, localized enhancement of vascular permeability, allowing curcumin to reach targeted regions directly without altering its physicochemical properties or requiring chemical modification. This approach preserves the intrinsic pharmacodynamics of curcumin while minimizing systemic exposure and off-target accumulation often observed with nanocarriers (Fortune et al., 2025). Collectively, these features suggest that the unmodified curcumin + FUS strategy provides a simpler and controllable means of enhancing brain bioavailability and achieving region-specific therapeutic effects compared with nanoparticle-mediated delivery.

Despite the strengths of this study, several limitations should be acknowledged. First, although neuroinflammatory assessment extended beyond astrocytic activation to include striatal Iba1 and IL-6 staining, no significant between-group differences were observed for these markers at the 4-week time point. Under the present experimental conditions, detectable neuroinflammatory changes were confined to astrocytic measures. In addition, inflammatory gene expression was not assessed. Future studies incorporating molecular-level analyses, such as quantitative polymerase chain reaction or enzyme-linked immunosorbent assay for pro-inflammatory mediators, may further clarify the anti-inflammatory mechanisms associated with curcumin treatment (Liu et al., 2025; Nebrisi, 2021).

Second, although improvements were observed in motor behavior, nigrostriatal TH immunoreactivity, and astrocytic activation, the study did not directly evaluate synaptic integrity. Immunohistochemical analysis of synaptic markers, such as synaptophysin or postsynaptic density protein 95 (PSD95), was not conducted. Consequently, it remains unclear whether the observed functional and histological changes are accompanied by alterations in synaptic structure. Prior experimental studies indicate that curcumin can influence synaptic-related proteins and dendritic spine density (González-Granillo et al., 2022; Li et al., 2025); therefore, inclusion of synaptic markers in future work would enable a more comprehensive assessment of how FUS-facilitated curcumin delivery affects synaptic alterations in Parkinsonian pathology.

Although EB extravasation and H&E staining demonstrated no evidence of excessive vascular leakage, hemorrhage, or parenchymal injury following repeated FUS exposure, the present study did not include molecular-level evaluation of BBB integrity. Advanced imaging techniques, such as magnetic resonance imaging (MRI), may provide additional information on BBB permeability dynamics after repeated sonication (De Maio et al., 2025). Moreover, immunohistochemical or immunofluorescent analyses of tight junction proteins, including claudin-5, occludin, and zonula occludens-1 (ZO-1), or assessment of endogenous serum protein leakage, such as IgG or fibrinogen, could complement the current safety evaluation by addressing subtle or transient BBB alterations (Gandhi et al., 2022; Morse et al., 2019; Sheikov et al., 2008; Tsai et al., 2018).

In addition, the present study does not examine oxidative stress or mitochondrial function, which are closely linked to Parkinson’s disease progression (Imbriani et al., 2022), and have been implicated in the reported actions of curcumin in toxin-based models (Du et al., 2012; Song et al., 2016; Tripanichkul and Jaroensuppaperch, 2013). The study also did not evaluate neurotrophic factors, such as glial cell line–derived neurotrophic factor or brain-derived neurotrophic factor, nor related signaling pathways. Previous work in 6-OHDA models suggests that both curcumin treatment and ultrasound-based interventions may influence neurotrophic factor–associated pathways (Jin et al., 2022; Song et al., 2022; Yang et al., 2014). The absence of these measures limits interpretation of whether such mechanisms contribute to the observed functional and histological outcomes.

Finally, only male mice were included in this study. Male animals were selected to reduce variability related to cyclical sex-hormone fluctuations; however, accumulating evidence indicates that responses in 6-OHDA models, neuroinflammatory activation, BBB permeability, and curcumin pharmacokinetics may differ by sex, with females often exhibiting distinct response patterns compared with males (Conner et al., 2020; Dei Cas and Ghidoni, 2019; Ince et al., 2023; Moon et al., 2021; Pettorossi et al., 2011; Pinizzotto et al., 2022; Solarz et al., 2021). Therefore, the findings of the present study may not fully generalize to females, and future studies including both sexes and considering hormonal influences are needed.

## 5 Conclusion

This study demonstrates that FUS effectively enhances the brain delivery of unmodified curcumin and is associated with improvements in motor function, preservation of dopaminergic neurons along the nigrostriatal pathway, and modulation of astrocytic activation in a 6-OHDA mouse model of PD. Without relying on complex formulations, this approach offers a practical strategy for future translational applications.

## Data Availability

The raw data supporting the conclusions of this article will be made available by the authors, without undue reservation.
